# Genome-wide association study dissects genetic architecture underlying longitudinal egg weights in chickens

**DOI:** 10.1186/s12864-015-1945-y

**Published:** 2015-10-05

**Authors:** Guoqiang Yi, Manman Shen, Jingwei Yuan, Congjiao Sun, Zhongyi Duan, Liang Qu, Taocun Dou, Meng Ma, Jian Lu, Jun Guo, Sirui Chen, Lujiang Qu, Kehua Wang, Ning Yang

**Affiliations:** Department of Animal Genetics and Breeding, College of Animal Science and Technology, China Agricultural University, Beijing, 100193 China; Jiangsu Institute of Poultry Science, Yangzhou, Jiangsu 225125 China

**Keywords:** Longitudinal egg weights, GWAS, SNP-based heritability, Polygene, Chicken

## Abstract

**Background:**

As a major economic trait in chickens, egg weight (EW) receives widespread interests in breeding, production and consumption. However, limited information is available for underlying genetic architecture of longitudinal trend in EW. Herein, we measured EWs at nine time points from onset of laying to 60 week of age, and conducted comprehensive genome-wide association studies (GWAS) in 1,534 F_2_ hens derived from reciprocal crosses between White Leghorn and Dongxiang chickens.

**Results:**

Egg weights at all ages except the first egg weight (FEW) exhibited high SNP-based heritability estimates (0.47 ~ 0.60). Strong pair-wise genetic correlations (0.77 ~ 1.00) were found among all EWs. Nine separate univariate genome-wide screens suggested 73 signals showing significant associations with longitudinal EWs. After multivariate and conditional analyses, four variants on three chromosomes remained independent contributions. The minor alleles at two loci exerted consistent and positive substitution effects on EWs, and other two were negative. The four loci together accounted for 3.84 % of the phenotypic variance for FEW and 7.29 ~ 11.06 % for EWs from 32 to 60 week of age. We obtained five candidate genes, of which *NCAPG* harbors a non-synonymous SNP (*rs14491030*) causing a valine-to-alanine amino-acid substitution. Genome partitioning analysis indicated a strong linear correlation between the variance explained by each chromosome and its length, which provided evidence that EW follows a highly polygenic nature of inheritance.

**Conclusions:**

Identification of significant genetic causes that together implicate EWs at different ages will greatly advance our understanding of the genetic basis behind longitudinal EWs, and would be helpful to illuminate the future breeding direction on how to select desired egg size.

**Electronic supplementary material:**

The online version of this article (doi:10.1186/s12864-015-1945-y) contains supplementary material, which is available to authorized users.

## Background

In poultry industry, egg weight has always been an economically important trait, and is usually regarded as the major breeding objective and research goal [1–4]. As a sensory feature at first glance, egg weight demonstrates a critical impact on consumption due to diverse preferences towards size worldwide [5, 6]. For breeders, egg weight has been reported to affect chick quality including hatching weight, fitness and performance [7, 8]. Notably, owing to the significant effect of hen age, egg weight shows consecutive increase during the whole laying period [9]. Eggs with extreme size in the later stage may obstruct automatic packing and consumption. Therefore, investigation of the genetic architecture underlying egg weights at different ages would have both economic and biological importance.

Multiple lines of evidence have suggested that egg weights are mainly determined by genetic factors and they all show considerable heritability estimates [4, 10, 11]. Currently, 77 quantitative trait loci (QTL) involved in egg weight, located on 17 different chromosomes, have been deposited in the AnimalQTLdb (http://www.animalgenome.org/cgi-bin/QTLdb/index). However, a majority of reported QTLs are mapped with wide confidence intervals by low-density linkage analysis in the past [12]. In general, it is very difficult to identify potential causal variants in QTL mapping studies, mainly due to the relatively small number of recombinants generated from two original parents in a limited number of generations [13]. To improve the precision of gene-level mapping, genome-wide association study (GWAS) based on linkage disequilibrium (LD) between SNPs and causal QTLs/genes is proposed as a more powerful approach identifying genetic links between phenotypes and genotypes [14, 15]. So far, two leading studies have successfully refined associated intervals affecting egg weight via moderate-density SNP chips [3, 16]. Recent advances in next-generation sequencing technologies enable the discovery of a large number of SNP markers as well as the development of high-density SNP platforms [17]. In chickens, the availability of 600 K Affymetrix Chicken SNP array could contribute to narrowing down candidate genomic segments and pinpointing several dominating causal variants.

Despite the great advantages of GWAS, most studies to date utilized only phenotypes measured at a single time point [3, 16, 18–20]. In practical genetic studies, some traits can be measured repeatedly over a period of time, and how to analyze these longitudinal data has received increasing attention [21–23]. Recently, the longitudinal GWAS has been proposed to assess whether some significant SNPs are associated with the process that a trait develops over time [21, 24]. Numerous studies have provided growing evidence that the longitudinal design could offer the opportunity for the identification of time-dependent and consistent loci [25, 26]. Moreover, joint analyses for measurements at multiple time points could increase the statistical power over cross-sectional approach [27, 28], owing to their effectiveness in incorporating the correlation structure of multiple measurements and alleviating the multiple testing burden [21, 29, 30]. Genome-wide assessment of longitudinal egg weight data would be beneficial to distinguish between genetic contributions reflecting constant and specific effects.

In the present study, we implemented univariate, multivariate and conditional GWASs using 600 K Affymetrix Chicken SNP array in a total of 1,534 F_2_ chickens with observations for egg weights at different ages. In addition, we examined the genetic architecture of egg weight by partitioning genetic variation according to chromosome. The main goals of our current work were to assess the feasibility of longitudinal data as an intriguing resource in GWAS, pinpoint associated loci and genes that contribute to the phenotypic variability and longitudinal trend in egg weight, and provide valuable insights into the genetic basis of longitudinal egg weights.

## Results

### Phenotype statistics and genetic parameters

Descriptive statistics for nine egg weight variables are presented in Table 1. We observed that egg weight displayed a curvilinear increase with advancing hen age. Particularly, all phenotypic values conformed to the normal distribution after rank-based inverse normal transformation, and these transformed values were used for all primary analyses. We quantified the additive genetic variations in liability to egg weights at different ages captured by all eligible GWAS markers. Univariate GCTA analyses revealed that all egg weight traits, except first egg weight (FEW), had highly heritable patterns (Table 2), and the highest SNP-based heritability estimate was found in EW36 (*h*_*snp*_^2^ = 0.60). Moreover, bivariate GCTA analyses indicated that egg weights at multiple ages were highly and positively interrelated, especially for egg weights at two neighboring time points. As the beginning of entire laying stage, FEW showed slightly lower genetic correlations with egg weights at the following ages (*r*_*g*_ < 0.90), compared with those among egg weights from 32 to 60 week of age (*r*_*g*_ > 0.90).

**Table 1:** Descriptive statistics for nine egg weights in the F_2_ population

Trait (g)^a^	N	Mean	SD	CV (%)	Min	Max
FEW	1,477	35.80	3.86	10.80	25.00	49.72
EW32	1,473	45.81	3.49	7.62	24.99	57.23
EW36	1,464	47.44	3.83	8.06	37.07	62.20
EW40	1,476	48.66	3.78	7.77	38.80	64.55
EW44	1,420	49.44	4.06	8.22	37.68	63.82
EW48	1,226	51.40	4.14	8.06	39.11	65.76
EW52	1,225	51.79	4.44	8.56	36.94	69.85
EW56	1,348	53.05	4.43	8.36	39.70	67.74
EW60	1,336	53.13	4.53	8.53	38.27	71.71

*N* number of samples, *Mean* arithmetic mean, *SD* standard deviation, *CV* coefficient of variance, *Min* minimum, *Max* maximum

^a^FEW = first egg weight; EW32, EW36, EW40, EW44, EW48, EW52, EW56, EW60 = egg weight per four weeks from 32 to 60 week of age

**Table 2:** Summary of genetic analysis for egg weights at different ages

Trait^a^	FEW	EW32	EW36	EW40	EW44	EW48	EW52	EW56	EW60
FEW	0.36 (0.04)	0.89 (0.04)	0.91 (0.04)	0.79 (0.05)	0.87 (0.05)	0.82 (0.05)	0.86 (0.05)	0.77 (0.06)	0.80 (0.05)
EW32	0.53	0.59 (0.04)	1.00 (0.01)	0.98 (0.01)	1.00 (0.01)	0.99 (0.01)	0.95 (0.02)	0.94 (0.02)	0.93 (0.02)
EW36	0.48	0.78	0.60 (0.04)	0.97 (0.01)	0.99 (0.01)	0.99 (0.01)	0.96 (0.02)	0.94 (0.02)	0.95 (0.02)
EW40	0.44	0.78	0.76	0.54 (0.04)	1.00 (0.01)	0.98 (0.01)	0.96 (0.02)	0.97 (0.02)	0.96 (0.02)
EW44	0.44	0.71	0.7	0.74	0.47 (0.04)	0.99 (0.01)	0.97 (0.02)	0.99 (0.02)	0.99 (0.02)
EW48	0.43	0.74	0.72	0.76	0.74	0.56 (0.05)	0.98 (0.01)	1.00 (0.02)	1.00 (0.01)
EW52	0.45	0.71	0.71	0.74	0.72	0.78	0.56 (0.05)	1.00 (0.01)	0.99 (0.01)
EW56	0.39	0.67	0.67	0.72	0.7	0.75	0.76	0.49 (0.05)	1.00 (0.01)
EW60	0.41	0.68	0.67	0.71	0.69	0.74	0.76	0.76	0.53 (0.05)

Diagonal: heritability estimates. Lower triangle: phenotypic correlations. Upper triangle: genetic correlations. Standard errors of the estimates are in parentheses

^a^FEW = first egg weight; EW32, EW36, EW40, EW44, EW48, EW52, EW56, EW60 = egg weight per four weeks from 32 to 60 week of age

### Identification of candidate loci by GWAS

**Fig. 1:**
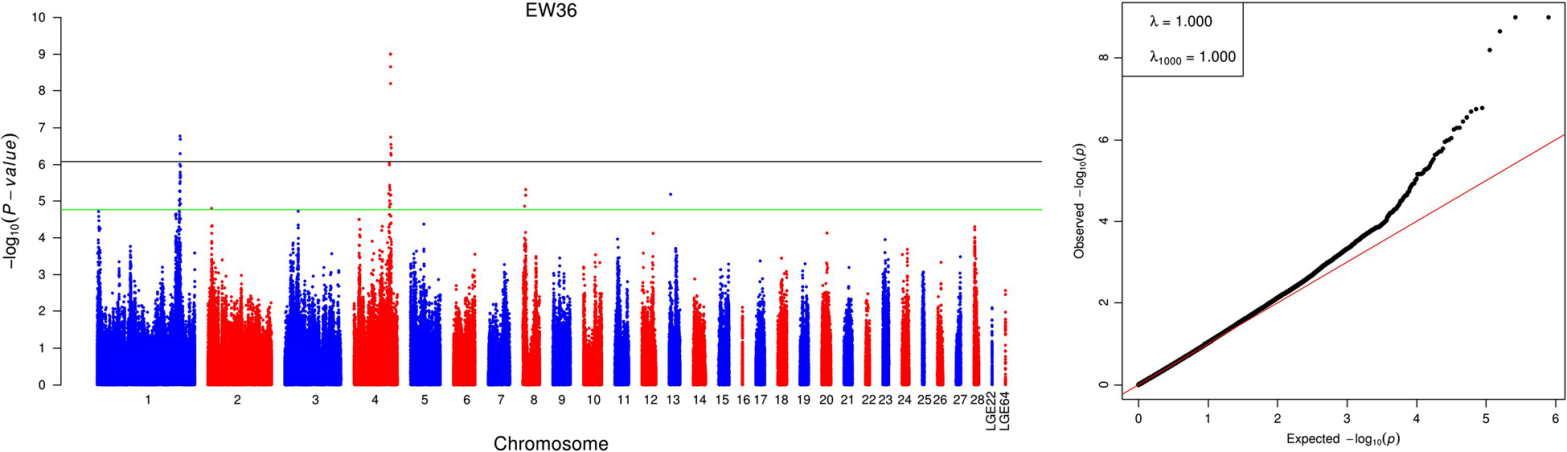
Manhattan plot (left) and quantile-quantile plot (right) of the observed *P*-values for egg weight at 36 weeks of age (EW36). The Manhattan plot indicates -log_10_ (observed *P*-values) for genome-wide SNPs (y-axis) plotted against their respective positions on each chromosome (x-axis), and the horizontal green and black lines depict the genome-wide suggestive (1.69 × 10^−5^) and significant (8.43 × 10^−7^) threshold, respectively. For quantile-quantile plot, the x-axis shows the expected -log_10_-transformed *P*-values, and the y-axis represents the observed -log_10_-transformed *P*-values. The raw and adjusted genomic inflation factors (λ and λ_1000_) are shown on the top left in the QQ plot

As a preliminary, we conducted nine separate association tests using univariate method for longitudinal egg weights. In total, 73 genome-wide significant SNPs located on chromosome 1, 3 and 4 (GGA1, GGA3 and GGA4) were successfully identified for these nine traits (Additional file 1: Table S1). These excess signals of association were entirely attributable to two chromosomal regions (GGA1: 167.4 ~ 174.4 Mb and GGA4: 73.1 ~ 77.2 Mb) and a single locus (*rs316497033* on GGA3). Of all significant association signals, 45 SNPs were responsible for only one trait and other 28 hits affected multiple phenotypes. In particular, four loci around *rs14491030* were found to be implicated in all egg weights except FEW. The global view of the putative *P-*values for all SNPs affecting EW36 is given in Fig. 1, and the Manhattan and quantile-quantile (QQ) plots for the remaining traits are provided in Additional file 2: Figure S1. The genome-wide discovery analyses yielded a small genomic inflation factor (λ) for each egg weight, ranging from 0.981 to 1.014. After adjustment by sample size, the standardized λ_1000_ values varied from 0.987 to 1.010. These results indicated negligible inflation of the observed genome-wide association signals caused by population stratification. Notably, moderate deviations within the upper tail of the distribution were found in all QQ plots, in spite of stringent quality control and inclusion of up to top five PCs. Thus, the departure due to a great number of weakly associated SNPs was indicative of polygenic inheritance.

In total, we obtained 270 genome-wide suggestive loci from nine independent univariate analyses. To enhance the statistical power, a joint GWAS analysis of nine traits was performed by fitting these SNPs into a multivariate model. Consequently, a total of seven significant hits on GGA1 and GGA4 provided compelling evidence for associations with longitudinal egg weights (Additional file 1: Table S2). We then performed stepwise conditional GWASs to prioritize separately associated SNPs owing to the potentially strong linkage disequilibrium (LD) between neighboring variants. After two-round adjustment in the multi-traits GWASs, two significant SNPs, *rs314058619* on GGA1 and *rs14491030* on GGA4, were uncovered to be independent signals. Meanwhile, another locus, *rs14916609* (*P*_*adjust*_ = 8.15 × 10^−5^) on GGA1, survived the genome-wide suggestive threshold of multivariate model. Considering that *rs314058619* and *rs14916609* are separated by 1.5 Mb and the pair-wise LD between them is relatively low at *D’ =* 0.37 and *r*^*2*^ *=* 0.09 (a LD block extends only for a short distance), we treated *rs14916609* as another separate signal. Taken together, we obtained three significantly independent loci associated with all egg weights from multivariate analysis.

**Fig. 2:**
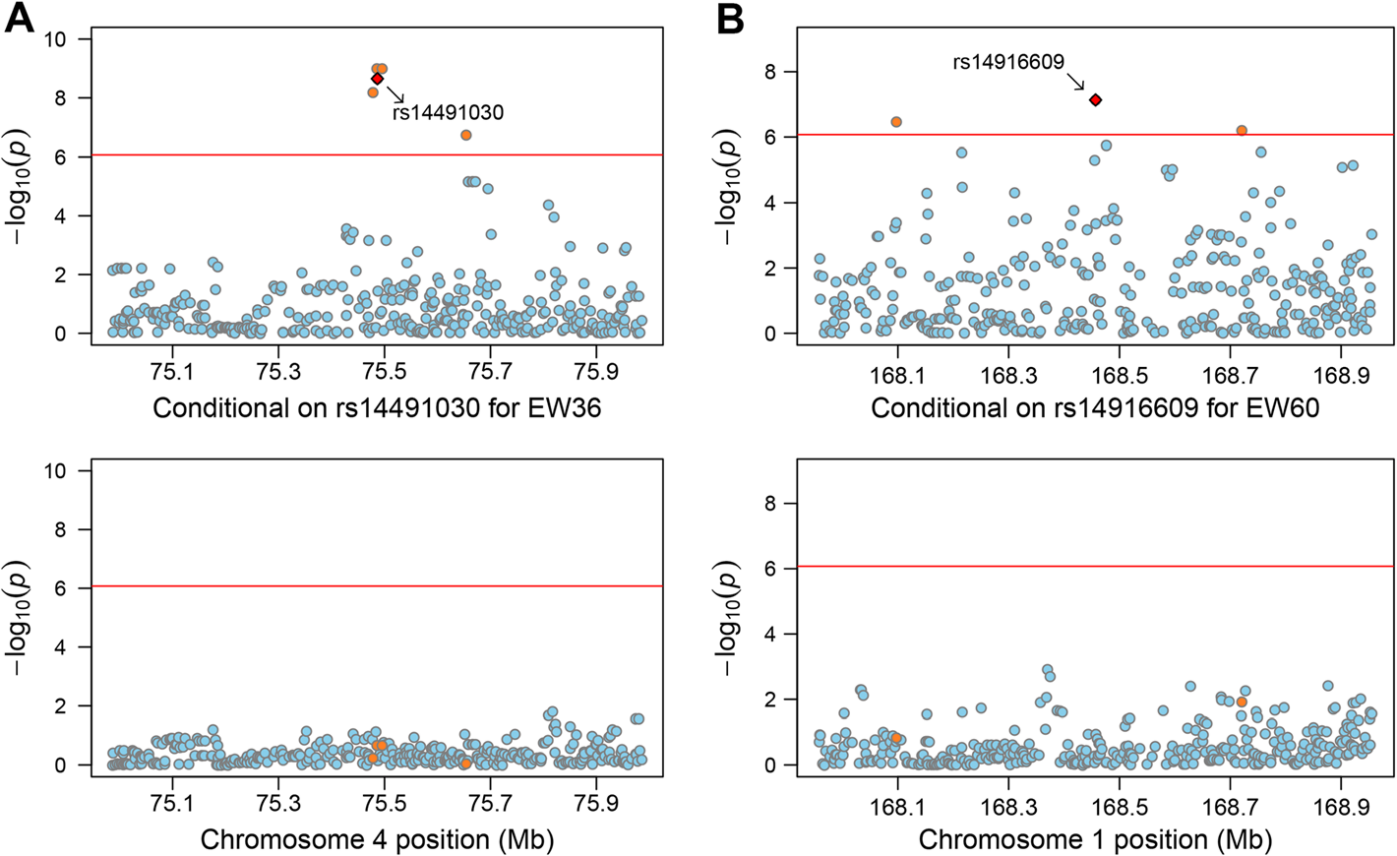
Regional association plots of two loci associated with EWs at 36 and 60 week of age (EW36 and EW60). For each plot, the -log_10_ (observed *P*-values) of SNPs (y-axis) are presented according to their chromosomal positions (x-axis). The horizontal red line depicts the genome-wide significance level (8.43 × 10^−7^). The significant SNP after univariate, multivariate and conditional GWASs is represented by a red diamond and is labeled by its rs number. **a** Regional association results for EW36 before (upper) and after (lower) conditioning on *rs14491030*. **b** Regional association results for EW60 before (upper) and after (lower) conditioning on *rs14916609*

After that, we repeated conditional analyses in the single-trait model, through including the three aforementioned alleles as covariates in an orderly manner (*rs314058619*, *rs14491030* and *rs14916609*). After two-step univariate conditional tests on *rs314058619* and *rs14491030*, the third SNP *rs14916609* was still significantly associated with all traits except FEW. This locus seemed to have independent effects on these traits, despite that it showed only suggestive significance inferred by two-round multivariate conditional GWASs. The finding further strengthened the validity regarding *rs14916609* as an independent hit. It should be noted that the significance levels of loci nearby the peak signals were substantially attenuated after adjusting for the three independent hits. In order to provide visual support of independent associations, we interrogated the signals in the 1.0 Mb genomic region surrounding the independent loci (500 kb each side). Since *rs14916609* and *rs14491030* affected more phenotypes, we illustrated their regional association plots for EW36 and EW60 to compare the difference of putative significance levels before (upper panel in Fig. 2) and after (lower panel in Fig. 2) conditioning on the two hits. Moreover, conditional analyses on three aforementioned hits revealed that an additional SNP *rs316497033* on GGA3 remained significantly involved in EW56. Therefore, we performed further analyses conditional on the genotype at *rs316497033* site and found no more genome-wide significant signals. Finally, the four loci were considered as independent associations with longitudinal egg weights after a set of three GWASs.

### Allelic contribution to phenotypic variation

**Fig. 3:**
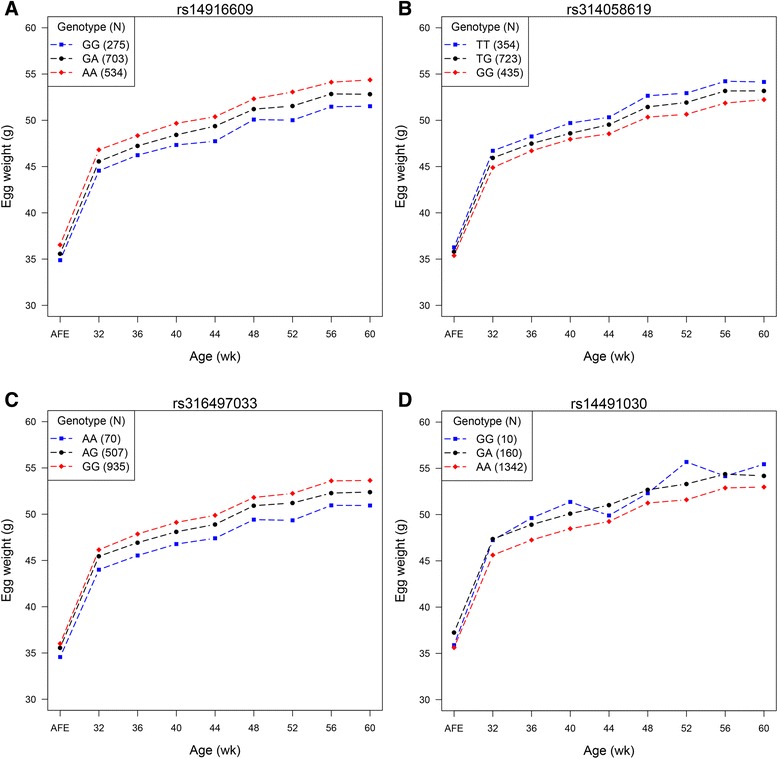
Consistent phenotypic differences contributed by four significant SNPs. Blue square, black circle and red diamond denote minor-allele homozygote, heterozygote and major-allele homozygote, respectively. Number of samples for each genotype is indicated in the top left corner. **a** Segregating phenotypes between three genotypes at *rs14916609*. **b** Segregating phenotypes between three genotypes at *rs314058619*. **c** Segregating phenotypes between three genotypes at *rs316497033*. **d** Segregating phenotypes between three genotypes at *rs14491030*

For the four resulting loci, the allelic substitution effects and phenotypic variance explained by them were estimated for nine egg weight traits (Table 3). The minor allele at each locus is treated as the effect allele according to the GEMMA definition. The effect alleles at two SNPs, *rs314058619* and *rs14491030*, were associated with the increase in egg weights at each time point, as opposite to other two variants. The genetic effects of the each allele at multiple ages had the same direction but slightly discordance, suggesting the presence of potential time-dependent impact. Through fitting the four variants into mixed model simultaneously, they together explained 3.84 % of the phenotypic variance for FEW and 7.29 ~ 11.06 % for EW from 32 to 60 week of age. Notably, *rs14491030* exhibited the largest allelic substitution effect on egg weight at each age. At nine different ages, substituting one copy of allele A by allele G at *rs14491030* site would cause 0.444 to 0.618 SD/allele increase in egg weight, in which the SD represents the standard deviation of EW at corresponding age. The corresponding phenotypic variance explained by this variant ranged from to 1.32 to 3.88 %. We compared the actual phenotypic difference between three genotypes at each locus and found that the three phenotypes showed significant segregation (Fig. 3). The comparative results revealed that the homozygote of effect allele possessed the highest or lowest egg weight at the same time point and the heterozygote are medium, suggesting that individual phenotype was more severely affected by homozygous effect allele. It should be noted that the homozygote of effect allele at *rs14491030* site showed unstable EWs with advancing hen ages compared with the heterozygote and the homozygote of alternative allele, and other three SNPs yielded concordant change trends for egg weights at multiple ages.

**Table 3:** Contributions of four associated SNPs to egg weights at different ages

SNP		*rs14916609*	*rs314058619*	*rs316497033*	*rs14491030*
Chromosome		GGA1	GGA1	GGA3	GGA4
Position (bp)^a^		168,456,799	169,930,974	23,254,004	75,486,534
EA/AA		G/A	T/G	A/G	G/A
EAF		0.41	0.47	0.21	0.06
FEW	beta (SE)^b^	−0.201 (0.051)	0.095 (0.047)	−0.129 (0.055)	0.366 (0.086)
	CPV (%)^c^	1.92	0.30	0.55	1.32
EW32	beta (SE)	−0.284 (0.051)	0.204 (0.046)	−0.132 (0.054)	0.601 (0.082)
	CPV (%)	3.75	2.10	0.57	3.88
EW36	beta (SE)	−0.259 (0.053)	0.135 (0.047)	−0.200 (0.055)	0.499 (0.083)
	CPV (%)	2.92	0.82	1.42	2.50
EW40	beta (SE)	−0.269 (0.051)	0.167 (0.046)	−0.204 (0.054)	0.567 (0.081)
	CPV (%)	3.45	1.49	1.68	3.51
EW44	beta (SE)	−0.283 (0.053)	0.189 (0.047)	−0.195 (0.056)	0.503 (0.085)
	CPV (%)	3.62	1.73	1.39	2.74
EW48	beta (SE)	−0.266 (0.057)	0.270 (0.051)	−0.190 (0.061)	0.455 (0.092)
	CPV (%)	3.00	3.43	1.22	2.03
EW52	beta (SE)	−0.303 (0.056)	0.258 (0.050)	−0.277 (0.060)	0.549 (0.092)
	CPV (%)	4.02	3.13	2.65	3.09
EW56	beta (SE)	−0.283 (0.054)	0.271 (0.048)	−0.317 (0.057)	0.453 (0.087)
	CPV (%)	3.63	3.54	3.38	2.12
EW60	beta (SE)	−0.286 (0.053)	0.241 (0.048)	−0.272 (0.057)	0.469 (0.085)
	CPV (%)	3.81	2.89	2.53	2.31

*EA* effect allele (minor allele), *AA* alternative allele (major allele), *EAF* effect allele frequency, *FEW* first egg weight, *EW32*, *EW36*, *EW40*, *EW44*, *EW48*, *EW52*, *EW56*, *EW60* egg weight per four weeks from 32 to 60 week of age

^a^Alleles are indexed to the forward strand of Galgal4 assembly

^b^Estimated allelic substitution effect per copy of the effect allele (EA) based on inverse-normal transformed scale under an additive model, expressed in SD unit/allele; SE = standard error of the beta

^c^CPV = contribution to phenotypic variance (%)

### Annotation of significant SNPs

Gene annotation of the four significant SNPs allowed us to identify genes related to longitudinal egg weights. Considering that the putative variants may be in high LD with a causal locus genuinely associated with phenotype, we inferred the LD block around the sentinel SNP using Haploview. Four blocks containing the four hits were identified and the detailed information is summarized in Table 4. To investigate the functional characteristics of the four blocks, we scanned these regions with Biomart system. Five different genes overlapping with the blocks were integrated into final gene set. The VEP tool revealed that the four SNPs were encompassed by four aforementioned genes, and three of them lies within introns and one resides in exon (Table 4). Notably, a SNP, *rs14491030* significantly present at the lowest minor allele frequency, is a non-synonymous mutation in exon 14 of *NCAPG* and mediates a valine-to-alanine amino-acid substitution (V674A). According to the putative SIFT score (0.74), the change of amino-acid was predicted as tolerated to protein structure and function.

**Table 4:** Genomic regions surrounding four significant SNPs

Tag SNP	Chromosome	Length of block (kb)	SNP number in the block	Nearest gene^a^	Ensembl gene ID	Location (kb)^b^
*rs14916609*	GGA1	57.9	19	*CAB39L*	ENSGALG00000017005	Intron
				*CDADC1*	ENSGALG00000017004	Downstream_18.2
*rs314058619*	GGA1	4.4	2	*FKHR (FOXO1)*	ENSGALG00000017034	Intron
*rs316497033*	GGA3	15.2	8	*KCNG3*	ENSGALG00000009919	Intron
*rs14491030*	GGA4	17.2	9	*NCAPG*	ENSGALG00000014425	Exon 14

^a^Identification of the gene according to Ensembl genes database 76

^b^Distance in kb of SNP for the gene

### Genome partitioning of genetic variation

**Fig. 4:**
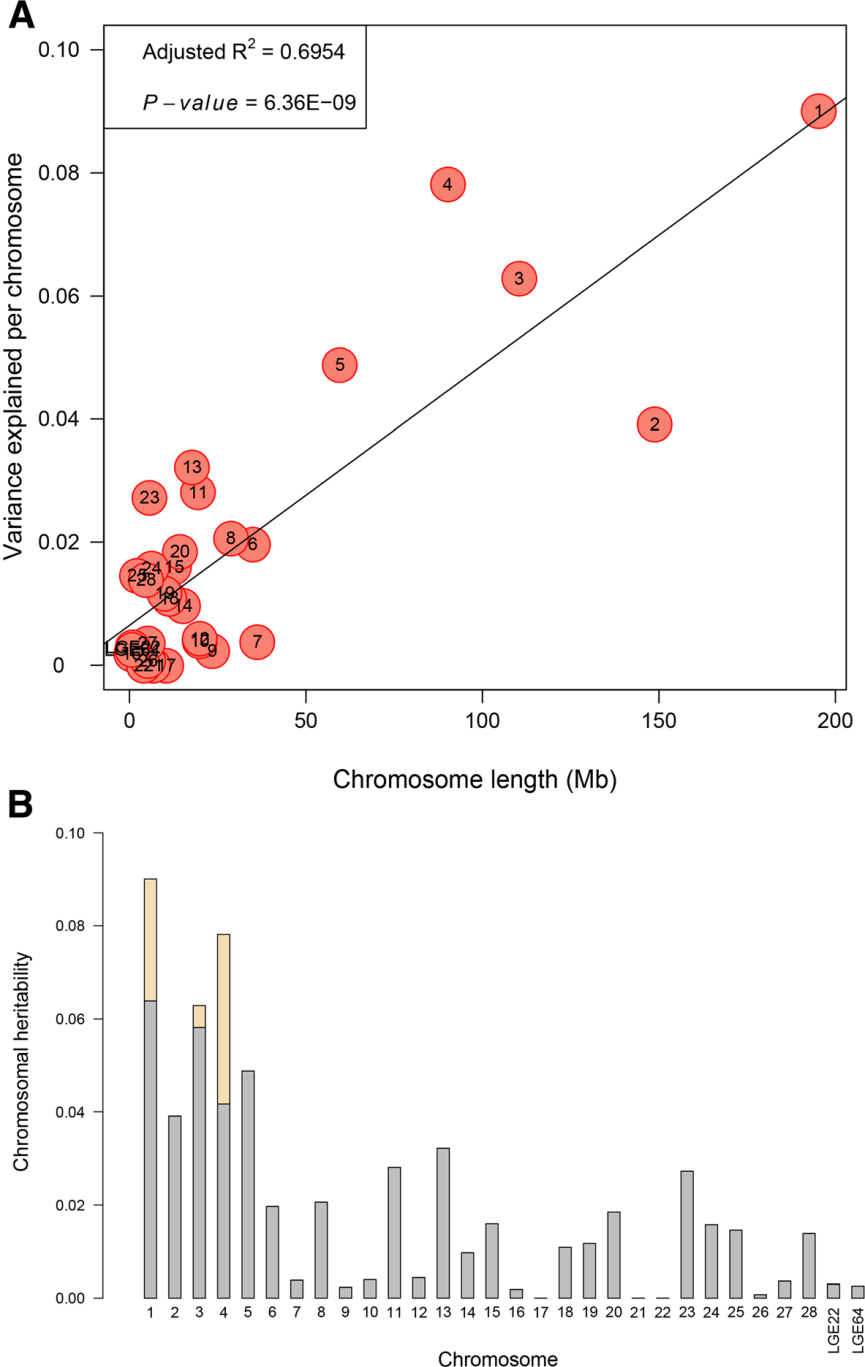
Genome partitioning for egg weight at 36 weeks of age (EW36) by joint analysis. **a** The estimated proportion of variance captured by each chromosome against its size. The characters in the circles are the chromosome numbers. **b** Contributions of GWAS SNPs partitioned by chromosome. The whole bars indicate the estimates of variance explained by each chromosome, in which the three wheat bars represent the same values by four resulting loci

We implemented an exploratory analysis through partitioning the genetic variation onto chromosome segments to further illustrate the genetic architecture of egg weight. Owing to relatively small sample size in the F_2_ population, parameter estimates for some traits in the joint model could not converge. However, the strong genetic correlations between nine egg weight phenotypes provided evidence that they had great potential to share a consistent genetic architecture. Therefore, we only exemplified the partitioning spectrum of EW36 due to its highest heritability. The estimates of variance contributed by each chromosome exhibited a strong linear relationship with the length of the chromosome for EW36 (*R*^*2*^ = 0.695, Fig. 4a), and no chromosome was found to show exceptional contribution. For three chromosomes, GGA1, GGA3 and GGA4, each of them explained more than 5.00 % of phenotypic variance, demonstrating higher genetic contributions than other chromosome segments (Fig. 4b). To quantify the effects of the four resulting variants on EW36, we fitted these SNPs as covariates and repeated the genome partitioning analysis. When compared with the results before adjustment, we found that the variance explained by GGA1 dropped from 9.01 to 6.39 % (Fig. 4b). The same estimate for GGA4 showed the largest decrease from 7.81 to 4.17 %. Particularly, a slight reduction in the estimated proportion of variance captured by GGA3 was found, and the estimates for other 27 chromosomes almost remained the same.

## Discussion

Although many genetic contributions to egg weight at a single time point have been unearthed by general GWAS approach [1, 3, 16], less is known about genetic architecture of longitudinal egg weights. To elucidate potential genetic loci that affect egg weight over time, we conducted comprehensive genome-wide association analyses for egg weights at multiple time points in an F_2_ segregating population, by utilizing the high-density 600 K SNP arrays. The current work not only provided a pioneering genome-wide association scan shedding light on longitudinal egg weight data, but also could deepen our understanding of genetic architecture underlying how egg weight develops over time. Through incorporating the associated loci into human-engineered breeding programs, we can select eggs with proper sizes to meet consumer needs, and help to predict the weight change in the following production.

Currently, conducting GWAS in an F_2_ population has been a routine approach to detect causal loci and genes [18, 20, 31]. GWAS approach is generally based on LD patterns in experimental populations, and the population structure is an important factor influencing the extent and range of LD [32]. Three previous studies [20, 33, 34] suggested that crossbred populations show a smaller extent and rapider decay of LD by distance as well as smaller haplotype block size than pure lines. The lower level of LD could enable that the associated SNPs are located in close proximity to the casual locus due to the tight linkage between them. Therefore, the F_2_ population and high-density array in our study would be beneficial for high-resolution mapping of genuinely causal loci.

All QQ plots displayed moderate leftward deflections of the observed distribution, suggesting the presence of numerous weakly associated variants. This phenomenon is often attributed to “spurious inflation”, and would be expected under polygenic architecture [35, 36]. Genetic analyses by GCTA tool revealed highly heritable patterns of egg weight. The high SNP-based heritability estimates showed that a substantial proportion of phenotypic variance could be captured by eligible GWAS SNPs, providing compelling support for polygenic model. Meanwhile, the strong genetic correlations between nine egg weight phenotypes indicated that these traits may share the similar genetic components or be influenced by some pleiotropic genomic regions [37, 38]. The results were also supported by the fact that the four independent SNPs exerted consistent effects on all egg weights. In poultry industry, maintaining good stability without significant increase after laying peak is another great concern [3, 4]. We previously conducted a genetic analysis for the increment traits of egg weight with advancing hen age [4], in which the increment of EW was defined as the difference between two EWs at neighboring ages. The low heritability estimates reflect that EWs at different ages may share similar genetic causes, and calculating the difference values may eliminate the same additive effects between them. Therefore, according to the two studies by us, how to improve the stability in egg weight seemed to be a considerable challenge by general genetic methods.

To further decipher the heritable architecture in egg weight, we partitioned the genetic variance onto different chromosomes based on estimated chromosomal GRMs [39, 40]. Notably, three macrochromosomes GGA1, GGA3 and GGA4 accounted for the largest genetic variance (>5.00 %), producing corroborative support for the GWAS results that four loci on them were identified as significant associations with egg weights. Moreover, a strong and positive linear correlation between variance explained by chromosomes and their length was found, suggesting the presence of a large number of genomic loci mediating weak effects [35]. The finding again consolidated the polygenic pattern in egg weight, and was consistent with other traits in animals [41, 42] and human [40, 43, 44]. The result reflected that longer chromosomes are likely to occupy more informative and effective markers, leading to a polygenic nature of egg weight [39]. Overall, despite that there were four SNPs with relatively large effects contributing to longitudinal egg weights, and our results demonstrated that many genetic variants each with a small effect widely spread across the whole genome. To pursue additional genetic loci with small or modest effect sizes, a larger population and high-throughput sequencing technology would be required.

Compared with some previous strategies leveraging only cross-sectional phenotype data [3, 16, 18–20], we took advantage of longitudinal egg weight data at nine time points. In total, we captured four promising loci with strong evidence for influencing egg weight over time. While we failed to detect some time-dependent variants, again suggesting that egg weight at different stages was likely to be affected by the same genetic loci. Regarding the four SNPs, only *rs14491030* was discovered to be responsible for all phenotypes except FEW via nine univariate analyses. This result indicated that the separate use of cross-sectional measurements might miss several loci which had weak effect sizes at one time point but large effects at other stages [26]. Cross-sectional surveys only identified time-dependent loci, but not provided insights into how such factors influenced traits over time [27, 45]. The longitudinal method was capable of permitting the investigation of genetic variants with consistent effects [26, 46]. In addition, univariate analysis for EW56 discovered a novel effective locus missed by multivariate method. The reason could attribute to smaller effect sizes of this mutation at other ages that had low power to uncover it. Moreover, it should be noted that multivariate test only used individuals with intact data records at each time point [29]. Considering that the number of analyzed individuals was 783 in multi-trait analysis, only one half of total sample size, thus some missing phenotypes at different ages may have a negative impact on the investigation of candidate variants [29, 47]. In consequence, to enhance the power detecting variants with consistent effects, we must enable more complete phenotypic information for each individual.

In total, four LD blocks tagged by the resulting SNPs were inferred, and contained five candidate genes of potentially functional relevance. Two SNPs (*rs14916609* and *rs314058619*) on GGA1 are segregated by 1.5 Mb and located in the intronic regions of *CAB39L* (calcium binding protein 39-like) and *FKHR* (forkhead box O1, also known as *FOXO1*), respectively. The *CAB39L* gene has been proposed to participate in the mTOR signaling pathway. It is increasingly apparent that mTOR signaling can affect most cellular functions as a central controller of cell growth (mass accumulation) and proliferation [48, 49]. Numerous studies have reported that *FKHR* is a member of *FOXO* subfamily and an important mediator of the insulin signaling pathway [50, 51]. This pathway could stimulate protein synthesis and cell growth by activation of mTOR [52]. For birds, the unfertilized yolk is a single cell (ovum), and the deutoplasm part will be gradually accumulated during oogenesis. The polymorphisms in the two genes may result in the change of egg weight through influencing the deposition of yolk and egg white.

A non-synonymous mutation (*rs14491030*) located within the *NCAPG* (non-SMC condensin I complex, subunit G) was unveiled in the current study. In chickens, many independent studies reported that multiple genomic segments containing this gene were identified to be implicated in both egg weight and body weight [1, 3, 16, 19, 20, 53]. However, a recent study revealed that another candidate gene *CCKAR* (cholecystokinin A receptor) located around 2.7 Mb away from *NCAPG* is mainly responsible for body weight [54], by establishing a 16-generation advanced intercross line (AIL) between a fast and a relatively slow growing strain. Because accumulated recombination events in the AIL are expected to provide better resolution for QTL mapping [55], *CCKAR* might be a more promising causal gene. Notably, the gene *NCAPG* has been suggested to be associated with many growth and weight traits in several livestock species [56–60]. Therefore, we could not ignore the potentially conserved function of *NCAPG* for body weight in chicken, owing to a particularly interactive relationship between egg weight and body weight [7, 61], i.e., the larger egg would cause higher hatch weight and the heavier chicken would produce larger eggs. We speculated that *NCAPG* gene may exert a pleiotropic effect involved in the two phenotypes simultaneously. Consequently, the impact on body weight of the gene for egg weight identified by us requires further investigation.

Another gene *CDADC1* (cytidine and dCMP deaminase domain containing 1) is located about 18.2 kb upstream of SNP *rs14916609*, but its function and mechanism is not yet clear. One previous study indicated that this gene may be an important factor regulating testicular development and spermatogenesis in human [62]. Due to its function on reproductive performance, we suspected that the gene may highlight a potential role on oogenesis. On GGA3, a gene *KCNG3* (potassium voltage-gated channel, subfamily G, member 3) indexed by *rs316497033* encodes a subunit of the potassium voltage-gated channel. It is known to be mainly involved in cell volume and smooth muscle contraction [63], and these biological functions may reflect potential connection with changed egg weight due to different alleles.

## Conclusions

In summary, we performed univariate, multivariate and conditional GWASs for longitudinal egg weight data using high-density 600 K SNP arrays, and suggested that longitudinal analysis had higher power to dig out variants that influence phenotype variability over time. Our study provided evidence that egg weight appears to be highly heritable and polygenic, and shares the similar genetic determinants and mechanisms at different ages. Four significant loci and five candidate genes were detected with significant effect on egg weights. In particular, *NCAPG* may influence both egg weight and body weight simultaneously in a pleiotropic manner. These promising loci and genes could be helpful to engineer practical breeding programs and produce the desired egg size according to diverse consumption markets.

## Methods

### Resource population

An F_2_ resource population was developed by reciprocal crosses between White Leghorn (WL) and Dongxiang chickens (DX), representing a popular commercial breed and a Chinese indigenous strain, respectively. The two pure lines were maintained for about ten years at the experimental farm, and WL eggs weighed about 14 g more than DX eggs at the same age. Six WL males were mated with 133 DX females and six DX males were mated with 80 WL females, to generate 1,029 and 552 birds of the F_1_ generation, respectively. Then, 25 males and 407 females from WL/DX cross and 24 cocks and 235 hens from DX/WL cross were selected to produce the F_2_ population. A total of 3,749 F_2_ individuals (1,893 pullets and 1,856 cockerels) with full pedigrees originating from 590 full-sib families were created in the same hatch. The population was maintained in three-tier single-hen cages and reared in the same environment with feed and water *ad libitum* at the experimental farm of Jiangsu Institute of Poultry Science. Finally, to ensure sufficient phenotypic information and accurate pedigree, we chose 1,534 hens from 49 half-sib families and 550 full-sib families for SNP genotyping.

### Phenotypic measurements

To characterize the genetic architecture of egg weight developmentally, we collected egg weight data at nine different time points. Firstly, the first egg weight (FEW) was recorded when hens started laying, mainly considering that FEW is conceptually defined as weight of first egg for each hen, and it is very difficult to collect eggs on several consecutive days due to the physiologically unstable state of hens at onset of laying. Subsequently, egg weights (EW) were measured on two consecutive days per four weeks from 32 to 60 week of age, and the average for two days was regarded as the phenotypic value for each hen. Cracked, soft-shell and double-yolk eggs were not used in our study. Descriptive statistics were calculated with the MEANS procedure of the SAS software package (SAS Institute Inc., 2006) using all available records. For traits deviating from normality, we conducted the rank-based inverse normal transformations (INTs) using SAS ahead of association tests [64]. Briefly, the procedure involves first transforming all observations to ranks and then converting these ranks to follow a standard normal distribution with a mean of 0 and an SD of 1. These transformed values were then used for the downstream analyses including GWAS discovery and heritability estimation.

### Genotyping, quality assurance and imputation

The whole blood samples were collected from brachial veins of chickens by standard venipuncture. Genomic DNA was isolated using standard phenol/chloroform extraction method and genotyped with the 600 K Affymetrix Axiom Chicken Genotyping Array (Affymetrix, Inc. Santa Clara, CA, USA). From an initial set of 580,954 SNPs [17], 7,883 SNPs with unknown genomic positions and 112 SNPs with redundant genomic coordinates were removed. Subsequently, first-pass quality control and genotype calling from the raw data in the form of CEL files were implemented with Affymetrix Power Tools v1.16.0 (APT) software using the Axiom GT1 algorithm. Only samples with dish quality control (DQC) of 0.82 or better and call rate > 97 % were included into the downstream analyses. An R script supplied by Affymetrix was run to compute the SNP QC metrics and filter out individual SNPs falling below given thresholds. All parameters were set to the default values recommended by Affymetrix. After these QC steps, 1,512 samples and 532,299 SNPs remained. In addition, we excluded 6,402 SNPs on sex chromosomes considering that current statistical methods are more powerful to detect the associations between phenotypes and autosomal genotypes. To improve the power of association analyses, we dropped 67,330 SNPs with minor allele frequency (MAF) < 5 % and 22,700 SNPs deviating from Hardy-Weinberg equilibrium (HWE) test *P* < 1 × 10^−6^ using the PLINK v1.90 package [65]. Some sporadic missing genotypes were imputed using the BEAGLE v4.0 procedure [66], then only SNPs with imputation quality score *R*^2^ > 0.5 underwent the next analyses step. Finally, a total of 1,512 samples and 435,867 SNPs were eligible for inclusion in the following GWASs.

### Association analysis

Prior to GWAS, we conducted a principal component analysis (PCA) to eliminate spurious associations due to the presence of potential cryptic relatedness or hidden population stratification. Considering that clusters of highly correlated SNPs may distort the resulting PCs, we first pruned the full SNP set to 41,130 independent SNPs via the *--indep-pairwise 25 5 0.2* command (PLINK), and then calculated top five PCs as covariates into the mixed model. To establish proper thresholds for genome-wide suggestive and significant associations, we corrected for multiple testing using the simpleM method [67] accounting for linkage disequilibrium (LD) relationships among SNPs. Using simpleM we estimated the effective number of independent tests as M_eff_ = 59,308, thus the genome-wide suggestive and significant *P*-values were 1.69 × 10^−5^ (1.00/59,308) and 8.43 × 10^−7^ (0.05/59,308), respectively.

We firstly performed univariate tests of association for SNPs having MAF ≥ 0.05 using an exact mixed model approach implemented in the GEMMA v0.94 software [68]. The centered relatedness matrix was calculated by those independent SNPs for all cases. And then each SNP was tested for additive association with each trait by modeling the effects of genotypes and the additional covariates including top five PCs as fixed effects, and random polygenic effects as random effects, mainly considering that our samples were from highly structured populations with strong family relatedness. The manhattan plot and quantile-quantile (QQ) plot depicting -log_10_-transformed observed *P*-values were generated using the “gap” and “qqman” packages in R [69, 70]. We calculated the genomic inflation factor λ to judge the extent of false positive signals [71]. Furthermore, given that the λ becomes larger with increasing of sample size, we also estimated an adjusted inflation factor by standardizing to a sample size of 1,000 [72, 73]. The univariate linear mixed model for each SNP marker and two compute methods for λ are as follows:

1 $$ \mathbf{y}=\mathbf{W}\boldsymbol{\upalpha } +\mathbf{x}\beta +\mathbf{u}+\boldsymbol{\upvarepsilon} $$

2 $$ \uplambda =\frac{Median\left({T}_i^2\right)}{0.455} $$

3 $$ {\uplambda}_{1000}=1+\frac{\uplambda -1}{n}\bullet 1000 $$

for the equation (1), **y** is an *n* × 1 vector of phenotypic values for *n* individuals, **W** is an *n* × *c* matrix of covariates (fixed effects, i.e., top five PCs) including a column vector of 1, **α** is a *c* × 1 vector of corresponding coefficients including the intercept, **x** is an *n* × 1 vector of marker genotypes at the locus tested, *β* is the corresponding effect size of the marker and all effects are reported for the minor allele in each marker, **u** is an *n* × 1 vector of random polygenic effects with a covariance structure as **u** ∼ N(0, **K**V_*g*_), where **K** represents a known *n* × *n* genetic relatedness matrix derived from SNP markers and V_*g*_ is the polygenic additive variance, and **ε** is an *n* × 1 vector of random residuals with **ε** ∼ N(0, **I**V_*e*_), where **I** is an *n* × *n* identity matrix, and V_*e*_ is the residual variance component. We used the Wald test statistic ${\mathrm{F}}_{\mathrm{Wald}}={\widehat{\beta}}^2/\mathrm{V}\mathrm{a}\mathrm{r}\left(\widehat{\beta}\right)$ for each SNP to test the null hypothesis *β* = 0, where the best linear unbiased estimate (BLUE) of *β* and the corresponding sampling variance $Var\left(\widehat{\beta}\right)$ are obtained by solving the mixed model equations (MME) based on estimated V_*g*_ and V_*e*_.

In the formula (2), *T*_*i*_^2^ is the estimated Wald statistic and asymptotically distributed as a chi-square with 1 degree of freedom under the null hypothesis, and usually called observed *χ*^2^ statistics, and 0.455 is the expected median of the standard *χ*^2^ distribution under the null hypothesis of no association. For the formula (3), *n* is the sample size and λ is the estimate from the total samples.

Similarly, we applied a multivariate association analysis that directly models nine measurements on an individual [29], to capture genetic variants affecting the longitudinal egg weights consistently over time. Considering that insignificant SNPs from univariate analyses are not likely to exceed the significance level of multivariate test and handling all SNPs simultaneously would increase computational burden, thus we only analyzed those suggestive SNPs from the univariate results in a multivariate model. For each SNP marker, a multivariate linear mixed model could be fitted in the following form:

4 $$ \mathbf{Y}=\mathbf{W}\mathbf{A}+\mathbf{x}{\beta}^T+\mathbf{G}+\mathbf{E} $$

where **Y** is an *n* by *d* matrix of *d* phenotypes for *n* individuals, **W** = (**w**_**1**_,⋯,**w**_**c**_) is an *n* × *c* matrix of covariates (fixed effects, i.e., top five PCs) including a column of 1 s, **A** is a *c* by *d* matrix of corresponding coefficients including the intercept, **x** is an *n*-vector of marker genotypes, *β* is a *d* vector of marker effect sizes for the *d* phenotypes. It should be noted that **G** is an *n* by *d* matrix of random effects with **G** ~ MVN_*n*×*d*_(0, **K**, **V**_*g*_) where **V**_*g*_ is a *d* by *d* symmetric positive definite matrix of genetic variance component, and **E** is an *n* by *d* matrix of residual errors with **E** ~ MVN_*n*×*d*_(0, **I**, **V**_*e*_) where **V**_*e*_ is a *d* by *d* symmetric positive definite matrix of residual variance component (**K** and **I** are the same in the two models). MVN_*n*×*d*_(0, **V**_1_, **V**_2_) denotes the *n* × *d* matrix normal distribution with mean 0, row covariance matrix **V**_1_ (*n* by *n*) and column covariance matrix **V**_2_ (*d* by *d*).

Owing to the different number of statistic tests, we further adjusted the significance level in multivariate testing accounting for the nine independent inquiries in usual single-phenotype strategy. For the multivariate model, we corrected the genome-wide suggestive and significant cut-offs as 1.52 × 10^−4^ and 7.59 × 10^−6^ through multiplying the raw thresholds by the number of phenotypes [29].

Notably, a genomic region containing a cluster of neighboring SNPs in strong LD is usually associated with a phenotype for high-density array. To demarcate independent association signals across the putative regions, we run stepwise conditional analyses both in multiple and single models, through fitting the genotypes (coded as 0, 1 or 2 alleles) at the strongest signal identified by the multivariate analysis as covariates [74]. The process was repeated until no SNP reached genome-wide significance threshold.

### Estimation of variance explained

For egg weight at each time point, univariate restricted maximum likelihood (REML) implemented in GCTA v1.24 program [75] was performed to estimate the heritability explained by the eligible SNPs (*h*_*snp*_^2^) for GWAS using the same inverse-normal transformed EW values. We also quantified the pair-wise genetic and phenotypic correlations among egg weights at multiple time points with the bivariate mixed model [76]. A genetic relationship matrix (GRM) was derived from all genotyped SNPs on autosomes and two linkage groups, and top five PCs calculated by the GCTA tool were included as covariates to account for the potential population structure. For those genome-wide significant SNPs, we estimated the phenotypic variance contributed by these associated loci in the following mixed linear model:

5 $$ \mathbf{y}=\mathbf{X}\mathbf{b}+{\mathbf{g}}_{\mathbf{G}}+\mathbf{e} $$

where **y** is an *n* × 1 vector of phenotypic values for *n* individuals, **b** is a vector of fixed effects with its incidence matrix **X**, **g**_**G**_ is a vector of aggregate effects of all SNPs, and *Var*(**g**_**G**_) = **A**_**G**_σ_G_^2^ with **A**_**G**_ being the SNP-derived GRM and σ_G_^2^ being the additive genetic variance, **e** is a random residual term with **e** ∼ N(0, **I**σ_e_^2^) where σ_e_^2^ represents the residual variance and **I** represents an identity matrix. In this model, we partitioned σ_G_^2^ into the contributions of selected SNP loci (${\upsigma}_{{\mathrm{G}}_1}^2$) and other variants (${\upsigma}_{{\mathrm{G}}_2}^2$) of the whole genome, and the contribution to phenotypic variance (CPV) of selected SNPs is defined as CPV $\mathrm{C}\mathrm{P}\mathrm{V} = {\upsigma}_{{\mathrm{G}}_1}^2/{\upsigma}_{\mathrm{P}}^2$ with σ_P_^2^ being the phenotypic variance.

In addition, we partitioned the chicken genome into 28 autosomes and two linkage groups, and jointly estimated their contributions to phenotypic variance for traits of interest using the similar model to formula (5). Before that, we built the GRM for each chromosome and next fitted 30 GRMs simultaneously in a joint analysis [39, 77]. A regression analysis was done by R to evaluate the relationship between the variance explained by each chromosome and its length.

### Linkage disequilibrium analysis and genes identification

In general, GWAS does not distinguish a genuine causal locus from those statistically significant loci within a strong LD region. Therefore, in order to characterize potential candidate genes responsible for a trait, we conducted LD analyses and inferred the haplotype blocks containing peak SNPs by Haploview v4.2 [78]. A block is derived using the solid spine algorithm, and defined as that the first and last SNPs in a region are in strong LD (*D’* ≥0.8) with all intermediate SNPs, but the intermediate SNPs are not necessarily in LD with each other. Subsequently, we performed functional annotation on the significant SNPs and searched for candidate genes in the blocks based on the Galgal4 assembly, using Variant Effect Predictor (VEP) and Biomart tools supported by Ensembl [79, 80].

## Additional files

Additional file 1: Table S1. Summary of genome-wide significant SNPs with respect to nine egg weights by univariate GWAS. **Table S2.** GWAS SNPs identified by nine-trait multivariate analysis. (XLSX 19 kb) Additional file 2: Figure S1. Manhattan plots (left) and quantile-quantile plots (right) for first egg weight (FEW) and egg weights at 32, 40, 44, 48, 52, 56 and 60 wk of age (EW32 and EW40~EW60). The Manhattan plots indicate -log10 (observed P-values) for genome-wide SNPs (y-axis) plotted against their respective positions on each chromosome (x-axis), and the horizontal green and black lines depict the genome-wide suggestive (1.69 × 10-5) and significant (8.43 × 10-7) threshold, respectively. For quantile-quantile plots, the x-axis shows the expected -log10-transformed P-values, and the y-axis represents the observed -log10-transformed P-values. The raw and adjusted genomic inflation factors (λ and λ_1000) are shown on the top left in the QQ plot. (A) ~ (H) indicate egg weights at eight different wk of age, respectively. (PDF 842 kb)

## Abbreviations

GWAS: Genome-Wide Association Study
SNP: Single nucleotide polymorphism
QTL: Quantitative trait loci
AIL: Advanced intercross line
INT: Rank-based inverse normal transformation
LD: Linkage disequilibrium
REML: Restricted maximum likelihood
BLUE: Best linear unbiased estimate
MME: Mixed model equation
GRM: Genetic relationship matrix
MAF: Minor allele frequency
HWE: Hardy-Weinberg equilibrium
PCA: Principal component analysis
VEP: Variant Effect Predictor
