# Supplementary material for: Genome-wide association study dissects genetic architecture underlying longitudinal egg weights in chickens
Source: BMC Genomics. 2015 Oct 5;16:746. doi: 10.1186/s12864-015-1945-y (PMC4595193; doi:10.1186/s12864-015-1945-y)

A: plots for FEW

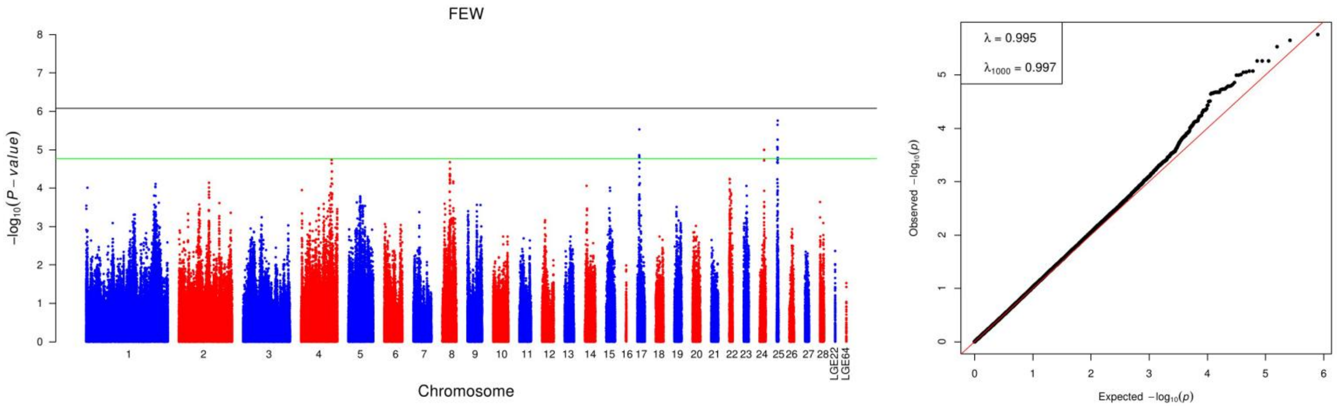

B: plots for EW32

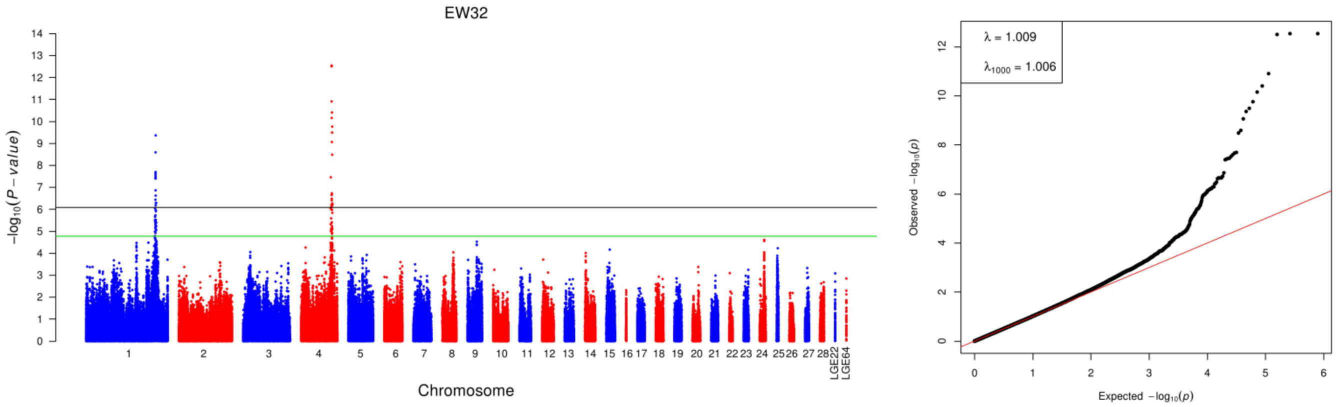

C: plots for EW40

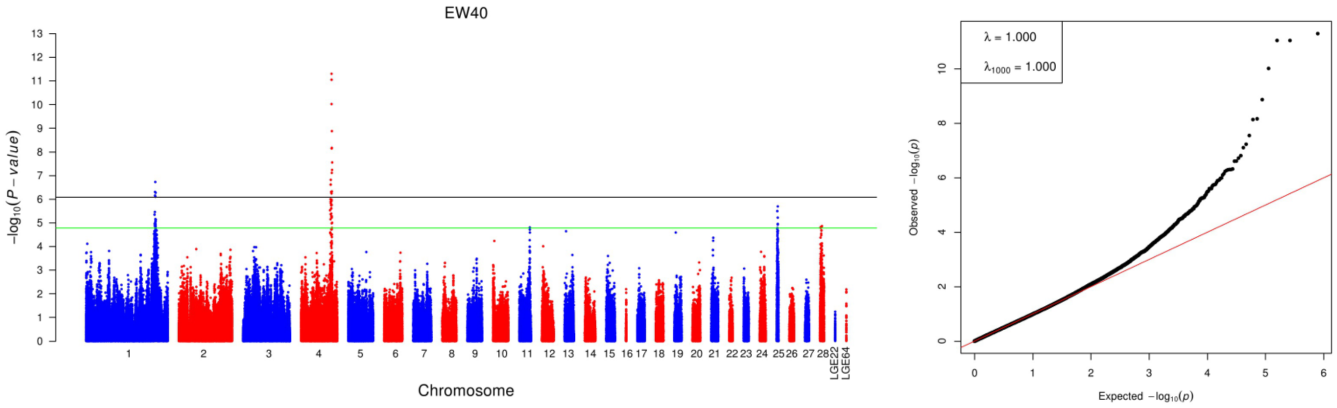

D: plots for EW44

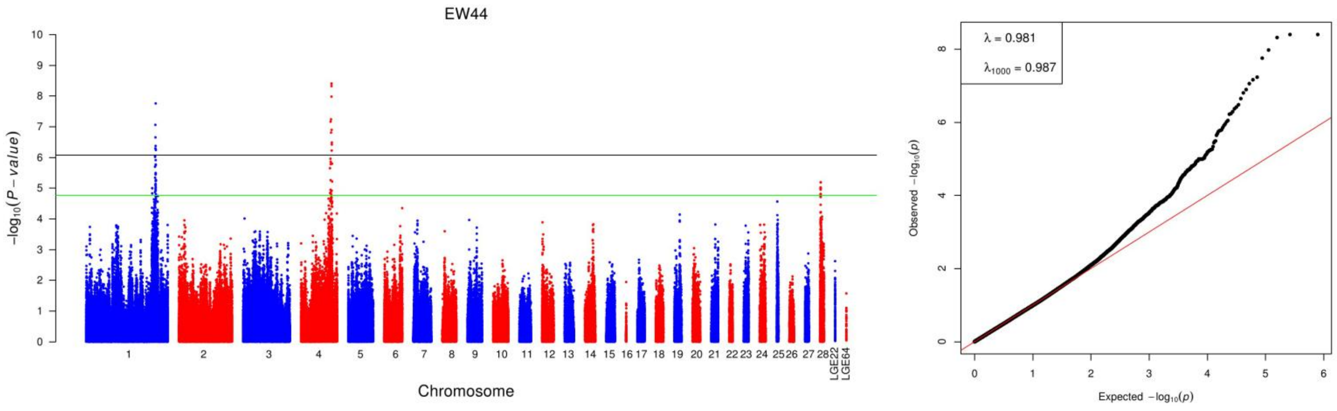

E: plots for EW48

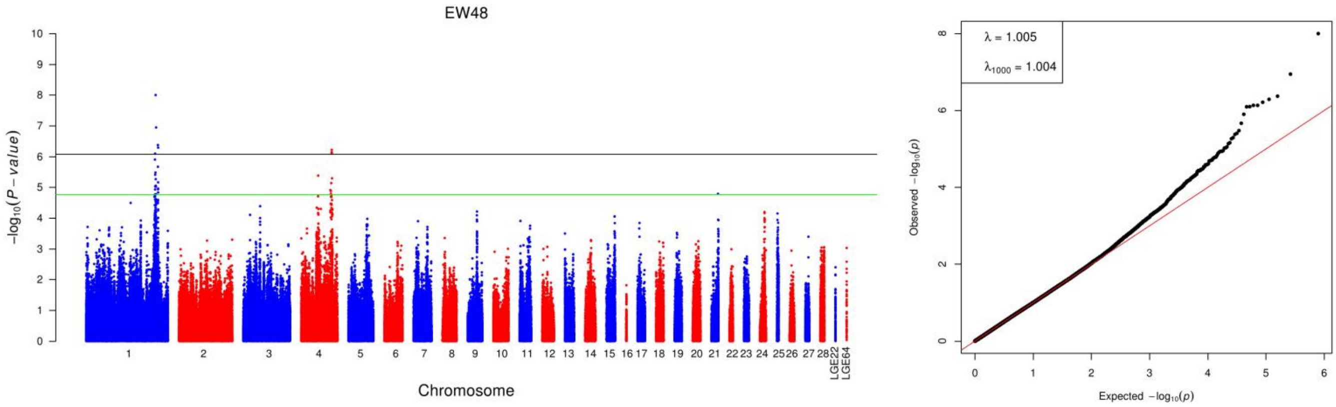

F: plots for EW52

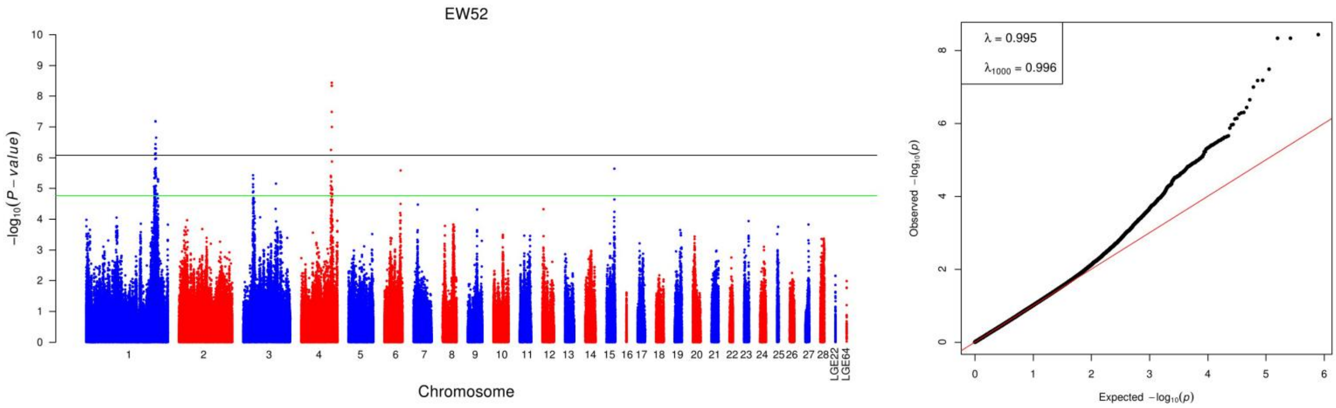

G: plots for EW56

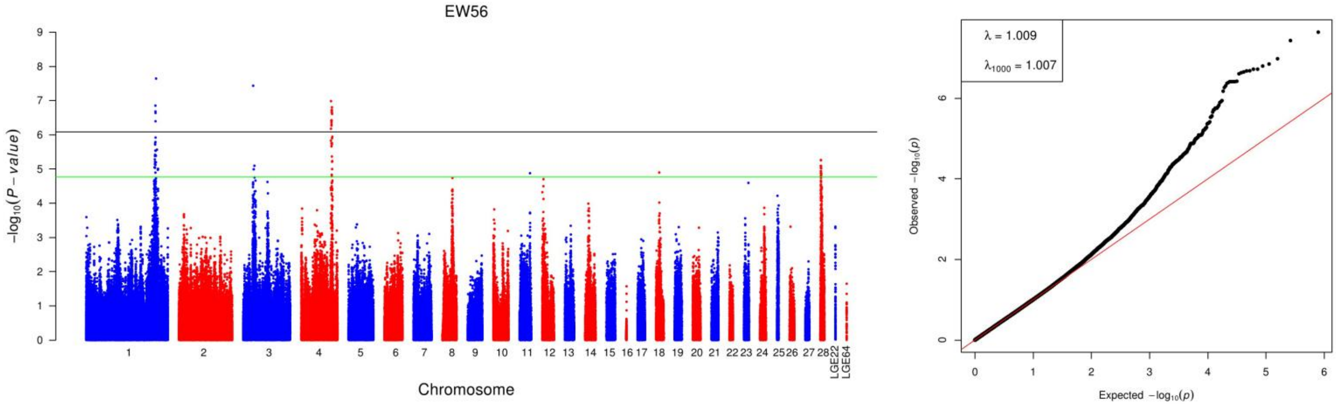

H: plots for EW60

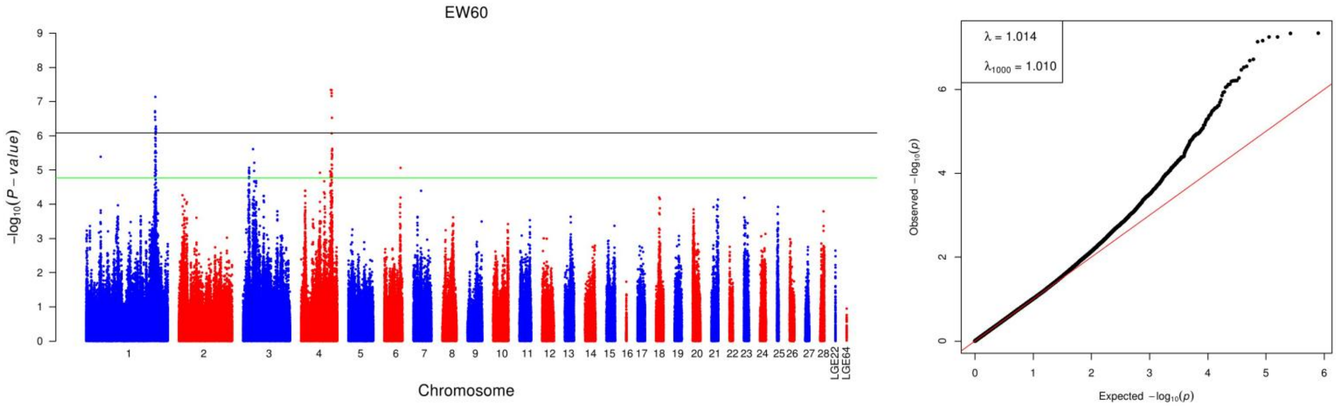

Supplement: Additional file 2: Figure S1. — Manhattan plots (left) and quantile-quantile plots (right) for first egg weight (FEW) and egg weights at 32, 40, 44, 48, 52, 56 and 60 wk of age (EW32 and EW40~EW60). The Manhattan plots indicate -log10 (observed P-values) for genome-wide SNPs (y-axis) plotted against their respective positions on each chromosome (x-axis), and the horizontal green and black lines depict the genome-wide suggestive (1.69 × 10^-5^) and significant (8.43 × 10^-7^) threshold, respectively. For quantile-quantile plots, the x-axis shows the expected -log10-transformed P-values, and the y-axis represents the observed -log10-transformed P-values. The raw and adjusted genomic inflation factors (λ and λ_1000) are shown on the top left in the QQ plot. (A) ~ (H) indicate egg weights at eight different wk of age, respectively. (PDF 842 kb) [file 12864_2015_1945_MOESM2_ESM.pdf]
